# Modern biology of extrachromosomal DNA: A decade-long voyage of discovery

**DOI:** 10.1038/s41422-024-01054-8

**Published:** 2025-01-03

**Authors:** Qing-Lin Yang, Yipeng Xie, Kailiang Qiao, Jun Yi Stanley Lim, Sihan Wu

**Affiliations:** 1 Children’s Medical Center Research Institute, University of Texas Southwestern Medical Center, Dallas, TX USA; 2 Department of Molecular Biology, University of Texas Southwestern Medical Center, Dallas, TX USA

**Keywords:** Cancer, Molecular biology

## Abstract

Genomic instability is a hallmark of cancer and is a major driving force of tumorigenesis. A key manifestation of genomic instability is the formation of extrachromosomal DNAs (ecDNAs) — acentric, circular DNA molecules ranging from 50 kb to 5 Mb in size, distinct from chromosomes. Ontological studies have revealed that ecDNA serves as a carrier of oncogenes, immunoregulatory genes, and enhancers, capable of driving elevated transcription of its cargo genes and cancer heterogeneity, leading to rapid tumor evolution and therapy resistance. Although ecDNA was documented over half a century ago, the past decade has witnessed a surge in breakthrough discoveries about its biological functions. Here, we systematically review the modern biology of ecDNA uncovered over the last ten years, focusing on how discoveries during this pioneering stage have illuminated our understanding of ecDNA-driven transcription, heterogeneity, and cancer progression. Furthermore, we discuss ongoing efforts to target ecDNA as a novel approach to cancer therapy. This burgeoning field is entering a new phase, poised to reshape our knowledge of cancer biology and therapeutic strategies.

## Introduction

**Fig. 1:**
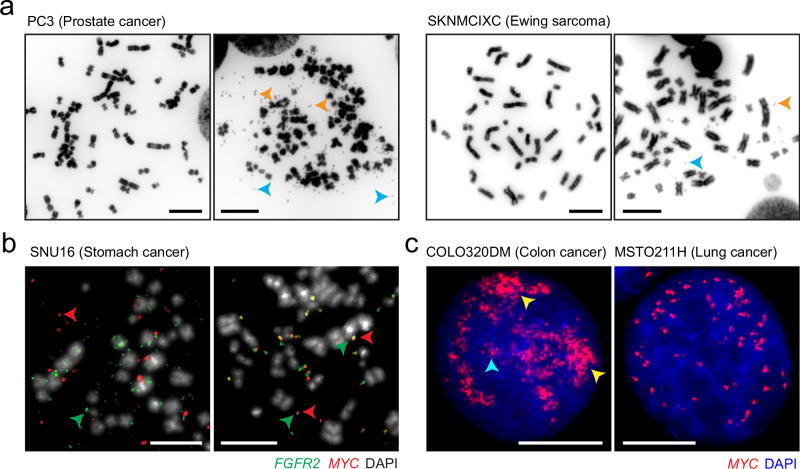
Heterogeneity of ecDNAs under the microscope. **a** Representative metaphase spreads from the PC3 prostate cancer cell line and the SKNMCIXC Ewing sarcoma cell line, showing a dramatic contrast in ecDNA counts between two individual cells. ecDNAs within the same metaphase spread can display double-minute (orange arrowheads) or singlet (blue arrowheads) morphology. **b** Two-color fluorescent in situ hybridization (FISH) to visualize ecDNA identity. Images were taken from two metaphase spreads from the SNU16 stomach cancer cell line. Two ecDNA species carrying different oncogenes, *FGFR2* (green) and *MYC* (red), may appear as individual particles (left) or as fusions (right), suggesting the ongoing evolution of ecDNAs. **c** Representative FISH images for *MYC* ecDNAs in interphase nuclei from COLO320DM colon cancer cell line and MSTO211H lung cancer cell line. *MYC* ecDNAs may cluster to form ecDNA hubs of different sizes (yellow arrowheads: large ecDNA speckle; cyan arrowhead: small ecDNA minim) in COLO320DM cells or appear as small to no aggregation in MSTO211H cells. Scale bars: 10 μm.

Despite the discovery of extrachromosomal DNA (ecDNA) in cancer dating back to 1965, when it was initially termed as double minutes (Fig. 1a),^1,2^ ecDNA was not fully acknowledged as a critical cancer driver until recent years. Thanks to a series of breakthrough findings that use contemporary genomic, genetic, imaging, and computation technologies, ecDNA’s biological functions and prevalence in cancer have become clear, bringing a conceptual advancement into cancer biology.^3^ Traditionally, ecDNA has been known as a carrier of oncogenes (Fig. 1b), as tumor-promoting genes and associated enhancers are often selectively enriched in ecDNAs.^4,5^ However, this definition has now been expanded, as oncogene-less ecDNAs carrying immunoregulatory genes or putative enhancers are coming into sight.^6–9^ While the function of oncogene-less ecDNAs remains largely undefined, their presence and amplification imply that these non-canonical ecDNAs may play a role in cancer development. It is worth mentioning that ecDNA differs from extrachromosomal circular DNA (eccDNA), which is much smaller in size and occurs in both cancer and non-cancerous cells across a wide range of eukaryotic organisms.^10–12^

One of the most distinguishable characteristics of ecDNAs is their lack of a centromere. Such an acentric nature allows ecDNAs to be inherited differently from chromosomes, leading to high-level amplification and heterogeneity. As demonstrated in Fig. 1a, which shows two images of the PC3 prostate cancer cell line, ecDNA may be absent in one cell but highly amplified in another cell. This, in turn, promotes massive gene expression and tumor progression.^13–15^ The circular shape also releases ecDNA from solving the “end-protection problem”^16^: While non-circular, acentric DNA fragments may also accumulate via asymmetric segregation, they will get progressively shortened or be eliminated through the DNA damage response pathway in the absence of a protective telomere. Furthermore, ecDNAs are thought to be more spatially mobile. Compared to chromosomes, the high copy number and relatively small size of ecDNAs may enable them to invade different nuclear territories, forming inter-molecular interactions and recombination sites that are otherwise restricted in chromosomes. This has evoked new thoughts about understanding the biological mechanisms and significance of ecDNA–ecDNA contacts,^17,18^ ecDNA–chromosome contacts,^19,20^ and even ecDNA–chromosome conversion.^21–24^

These unconventional genetic behaviors of ecDNAs have been linked to poor clinical outcomes. Patients with tumors harboring ecDNAs tend to have worse survival rates.^4,25–27^ Hence, ecDNA has emerged as a major obstacle in cancer therapy and is now recognized as one of the current Cancer Grand Challenges.^28^

In this review, we highlight the latest advancements that refine our understanding of ecDNAs and discuss plausible strategies to target them for treating advanced, ecDNA-driven cancers.

## Revisiting ecDNA biology

### ecDNA is a versatile template for transcription

ecDNA is a template for RNA transcription and it is armed with multiple mechanisms to boost oncogene outputs.

**Fig. 2:**
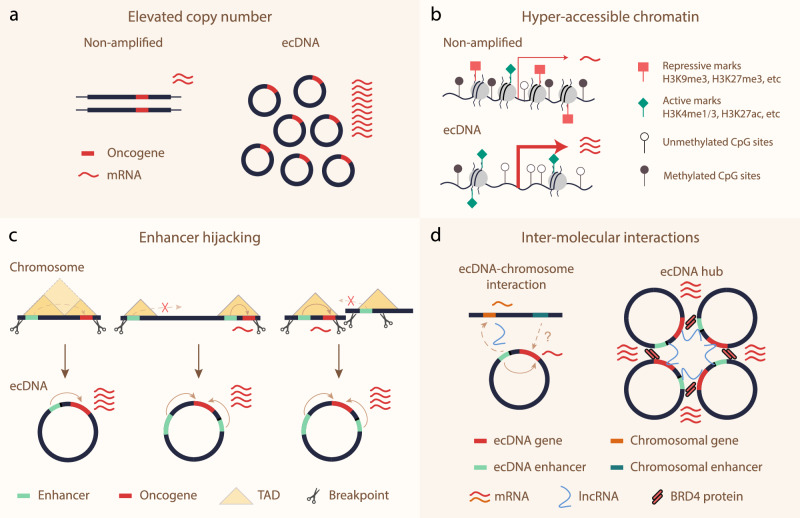
Mechanisms for enhanced expression of oncogenes on ecDNA. **a** Oncogene amplification through ecDNA produces more copies of oncogene, resulting in more transcripts than non-amplified chromosomal loci. **b** ecDNA chromatin is more accessible, with less repressive histone marks (H3K9me3 and H3K27me3) and repressive DNA methylation, and more active histone marks (H3K4me1/3 and H3K27ac) compared to non-amplified chromosomal loci. **c** ecDNA formation enables oncogene to hijack distal, non-canonical enhancers, either from the same topologically associated domain (TAD, left), a different TAD (middle), or a different chromosome (right). **d** ecDNA has profound interactions with chromosomes and other ecDNA molecules. ecDNA-borne nascent long non-coding RNA (lncRNA) coordinates enhancer–gene interactions between ecDNA–chromosome and ecDNA–ecDNA interactions. In addition, BRD4 proteins mediate ecDNA hub formation.

First, ecDNAs often exhibit high copy numbers (Fig. 2a). Within a single tumor cell, the number of ecDNAs can vary from none to several hundred (Fig. 1a).^14^ Allele analyses of whole-genome sequencing (WGS) and RNA sequencing have shown that ecDNAs may contribute to most of the transcripts of the amplified loci, pushing oncogene transcription to the top 1% of the transcriptome in some cases.^13^

How do ecDNAs achieve such a high copy number? Because ecDNA only replicates once during the S phase,^29^ the plausible explanation is the interplay between the random mitotic segregation of ecDNAs and the positive selection of cells expressing fitness genes at a reasonable level. When a cancer cell divides, post-replicated acentric ecDNAs are asymmetrically distributed into two daughter cells.^15,18^ If the tumor microenvironment favors the cell with a higher expression of an oncogene, the daughter cell with a higher ecDNA copy number acquires a proliferative advantage.^3,30^ This, in turn, increases the ecDNA copy number with each generation. Of course, the ecDNA copy number cannot elevate indefinitely, as excessive oncogenic signaling imposes cellular stress.^31^ While chromosome-based focal amplification mechanisms, such as the breakage-fusion-bridge (BFB) cycle, can generate long DNA repeats over extended periods,^32^ the rapid dynamics of ecDNA, facilitated by random segregation, enables ecDNA to attain a high and optimal copy number more rapidly.^14,15^

The chromatin status dictates whether a DNA template can be transcribed.^33^ In ecDNA, the chromatin is loosely packed compared to chromosomal DNA (Fig. 2b). Through assay for transposase-accessible chromatin with sequencing (ATAC-seq), ecDNA is found to possess typical mono-, di-, and tri-nucleosomes, which are the signature of accessible chromatin.^13^ In contrast, it lacks higher-order structures characteristic of compact heterochromatin, as indicated by the absence of long fragments in ATAC-seq reads.^13^ Further insights from chromatin immunoprecipitation followed by sequencing (ChIP-seq) and immunofluorescence experiments indicate that ecDNAs are enriched with active histone marks, such as H3K4me1/3 and H3K27ac, and are deficient in repressive histone marks like H3K9me3 and H3K27me3.^13^ Additionally, Nanopore single-molecule sequencing reveals a lower level of DNA methylation on gene promoters in ecDNAs, compared to those in native chromosomal regions and other ecDNA regions.^6^ This observation suggests increased promoter activities on ecDNA. Nonetheless, it remains unclear why the epigenetic landscape of ecDNA is uniquely regulated, especially compared to its chromosomal counterpart.

*Cis*-regulatory elements, such as enhancers, are critical components in the transcription circuit.^34^ If an ecDNA were formed as a DNA plasmid engineering process using genomic material, what configuration could maximize oncogene output? If ecDNA is made with only one DNA fragment, it would be logical to select the piece that contains both the oncogene and the adjacent enhancer (Fig. 2c, left panel). This is indeed the case, as enhancers surrounding an oncogene are often co-selected to be amplified in the same circular structure.^5,35^ Furthermore, the circularization of a DNA segment allows an oncogene to interact with distal enhancers by bringing them into proximity (Fig. 2c, middle panel).^35^

What if an ecDNA is built with multiple fragments during ligation? This process resembles some of the structural variation mechanisms in chromosomes. In ecDNA, the ligation substrates are the focally resected fragments from the chromosome, such as those derived from chromothripsis.^22,36^ Through this process, an oncogene on ecDNA can not only interact with distal enhancers that are several mega-bases away from its original chromosomal location,^6,37^ but also hijack enhancers from different chromosomes (Fig. 2c, right panel).^38^ Although it is unclear whether hijacking distal enhancers is more advantageous for maximizing transcriptional output compared to local ones, it likely offers flexibility for the oncogene on ecDNA to form versatile transcription circuits that can respond to alternative upstream signaling cues. In addition, co-amplification and even co-selection of local and distal insulators may be involved in building the transcriptional circuits in an ecDNA.^35^ Notably, the metaphor here by no means implies that there is an active process in formulating an ecDNA particle in cancer. What we observed from DNA structural analyses and chromatin conformation capturing assays (such as Hi-C) is the final outcome of random DNA ligation in the tumor cell and cellular selection by the tumor microenvironment.

All discussions so far have focused on a single ecDNA. However, as ecDNAs are often highly amplified, understanding how they interact through inter-molecular contacts is necessary to unravel the impact of ecDNA on the transcriptome in cancer. In eukaryotic cells, each chromosome occupies a distinct nuclear space, separate from adjacent chromosomes, called a “chromosome territory”.^39^ Such spatial organization limits inter-chromosomal contacts that could interfere with important intra-chromosomal interactions, although “kissing” between adjacent chromosomes has been observed.^40,41^ Chromosome 21, the smallest human chromosome, spans about 45 Mb.^42^ In contrast, a typical ecDNA is only 1 Mb.^6,13^ Does their small size allow ecDNAs to seep through the “gaps” among chromosome territories and interact with chromosomes? Recent studies have revealed that ecDNA–chromosome interactions regulate transcriptome.^19,20^ More specifically, enhancers on ecDNA can interact with promoters on chromosomes, which may promote the expression of targeted genes located on chromosomes (Fig. 2d, left panel).^19,20^ The nature of the targeted genes and the biological significance of ecDNA–chromosome interactions need further studies, especially whether such interactions confer any fitness to tumor development. This is a particularly challenging question to address because one ecDNA particle may have many different interaction sites on chromosomes among individual cells as estimated in a preprint study.^19^ Therefore, any bulk-cell genomic profiling approach will underestimate the actual effect size due to signal averaging and dilution. Implementing single-cell technologies here is essential. In addition, it remains unclear whether chromosomal enhancers can regulate gene expression on ecDNAs.

ecDNAs may spatially aggregate to form ecDNA hubs, permitting ecDNA–ecDNA interactions (Figs. 1c, left panel, 2d, right panel).^17,18^ ecDNAs within hubs exhibit higher levels of transcription compared to singleton ecDNAs within the same tumor cell.^17^ Such inter-ecDNA contacts likely occur through inter-molecular enhancer–promoter interaction bridged by BRD4, as demonstrated in the COLO320DM colon cancer cell line with *MYC* ecDNAs.^17^ However, the question of whether BRD4 is the universal ecDNA hub organizer warrants further investigation. Inter-ecDNA interactions are diverse. In some instances, dozens of ecDNAs aggregate to form large, micron-size ecDNA speckles (Fig. 1c, left panel, yellow arrowheads).^19^ Conversely, weaker ecDNA aggregation also occurs, with only a few ecDNA particles clustering in proximity to form ecDNA minim (Fig. 1c, left panel, cyan arrowhead).^19^ In some cases, ecDNAs may not interact (Fig. 1c, right panel), and the transcriptional activity of ecDNA-borne genes is similar to that of their chromosomal counterparts.^43^ Chromatin-associated RNA (caRNA) emerges as a key factor influencing the ability of ecDNA aggregation as suggested by a preprint study.^19^ Highly aggregated ecDNAs harbor an elevated level of ecDNA-borne, long non-coding caRNAs. Knockdown of the nascent transcript of *PVT1*, a long non-coding gene located near *MYC*, can disrupt *MYC* ecDNA speckles and decrease gene expression.^19^ More interestingly, ecDNA aggregation and ecDNA–chromosome interaction frequency show a positive correlation, with both being regulated by caRNAs.^19^ Further exploration is required to understand how commonly caRNAs are involved in the ecDNA interactome in cancer.

Finally, it is unclear how ecDNAs are spatially partitioned. Heterochromatin and euchromatin in chromosomes are spatially segregated in different nuclear compartments, with heterochromatin mainly organized around the nuclear periphery and nucleoli, while euchromatin is located in the interior of the nucleus.^44^ This spatial organization is tightly linked to transcription regulation.^45,46^ ecDNAs are enriched in accessible chromatin; and it is likely that transcriptionally active ecDNAs are organized in the interior space. However, as a peculiar DNA molecule that has broken many genetic laws, unbiased experimental evidence is required to understand the spatial compartmentation of ecDNAs.

### ecDNA-driven tumor heterogeneity: through the lens of evolutionary biology

**Fig. 3:**
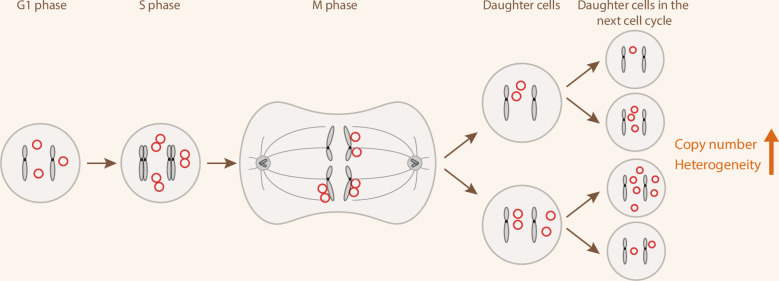
Non-Mendelian segregation of ecDNA during mitosis. ecDNAs replicate along with chromosomes in the S phase, appearing as double minutes. During mitosis, acentric ecDNAs attach to chromosomes and segregate randomly, resulting in unequal ecDNA copies in daughter cells. Multiple rounds of cell division increase ecDNA copy number and heterogeneity.

The most notorious function of ecDNA, perhaps, is that it gives rise to tumor heterogeneity due to unequal mitotic segregation. ecDNAs do not contain centromeres, as shown by both imaging and sequencing.^14,47^ Thus, ecDNAs cannot attach to the mitotic spindle during cell division. Instead, ecDNAs hitchhike with chromosomes to segregate into the daughter cells.^48^ As seen in live-cell microscopy and computational simulations, ecDNAs are distributed unequally to two daughter cells in every cell cycle, generating a pool of heterogeneous cancer cells with varying copies of ecDNAs (Fig. 3).^15,18,49,50^ In cancer types such as medulloblastoma, glioblastoma, and small cell lung cancer, asymmetric ecDNA segregation has been shown to drive rapid tumor evolution and confer adaptation to stress.^26,37,51^ Moreover, a recent study found that, through ecDNA–ecDNA interactions, ecDNAs of different sequences can co-segregate into the same daughter cell.^52^

**Fig. 4:**
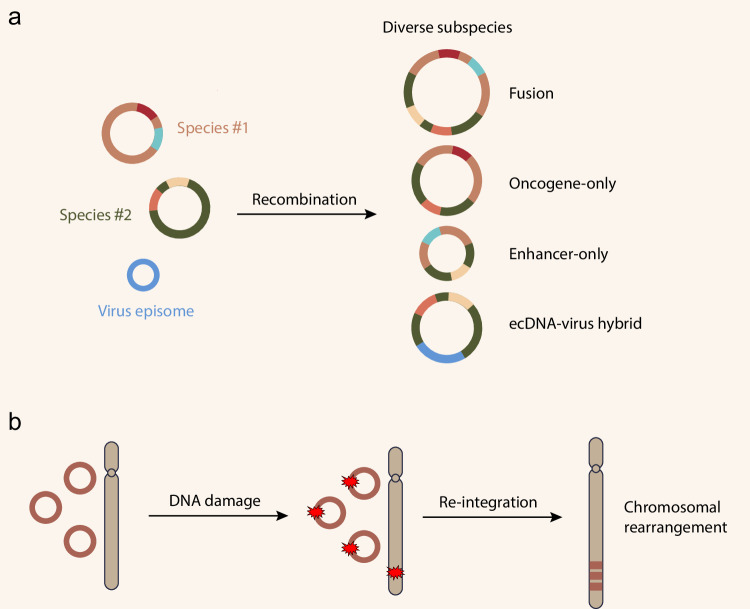
ecDNA structure heterogeneity. **a** Within a single cell, different species of ecDNAs co-exist and can undergo circular recombination, generating various subspecies of ecDNAs. ecDNA–virus hybrid was also observed, possibly due to recombination between ecDNA and virus episome. **b** ecDNA can reintegrate into chromosomes, leading to chromosomal rearrangement.

The complexity of the heterogenous ecDNA ecosystem extends beyond the copy number. The diversity in ecDNA species and structure adds another dimension to this complexity (Fig. 4a). If we define an ecDNA species by its signature identity (such as the oncogene it carries) and consider ecDNAs with the same signature identity but variable structures as subspecies, it has been found that multiple ecDNA species and subspecies can co-exist within a tumor population (Fig. 1b). Diverse ecDNAs can be characterized using DNA-targeted capturing and single-molecule technologies. For instance, by CRISPR-CATCH, ecDNA harboring different structural rearrangements can be separated by CRISPR-Cas9 linearization of DNA circles and subsequent pulse-field gel electrophoresis, enabling the sequencing for enriched ecDNAs to identify their heterogeneous structures.^6^ Nanopore sequencing, which provides long-read information for each ecDNA molecule, was also utilized to reveal diverse ecDNA structures at single-molecule resolution.^53^ These technologies allow us to explore the ecDNA landscape and identify ecDNA diversity, a critical component of intratumoral genetic heterogeneity.

Recently, ecDNAs with peculiar structures have been reported. For example, oncogene-less, enhancer-only ecDNAs have been identified.^5,6^ In cancer associated with episomal virus infection, such as human papillomavirus (HPV)-associated oropharyngeal cancer and HBV-associated liver cancer, part of the viral genome can integrate with the cancer genome to form virus-human hybrid ecDNAs (Fig. 4a).^8,54^ Considering that ecDNAs form novel intra- and inter-molecular interactions, it will be intriguing to study how diverse ecDNA species cooperatively regulate the cancer transcriptome and facilitate clonal expansion.

The existence of ecDNA subspecies suggests two possible mechanisms of their origins: (1) Diverse ecDNA subspecies were established at the moment of their creation. (2) An ecDNA species undergoes continuous evolution that gives rise to diverse ecDNA subspecies. While the first possibility is plausible, emerging evidence suggests that the ecDNA structure is unstable and is shaped throughout the disease progression, whose structural complexity may increase alongside.^7,53^ The use of parallel sequencing of extrachromosomal circular DNA and transcriptome at single-cell resolution (scEC&T-seq) allows us to infer the dynamics of ecDNA evolution.^53^ For example, in a neuroblastoma patient sample, many *MYCN* ecDNA subspecies were found in different clones. These subspecies share one common structure but exhibit diversity in other structural variations. Further structural analysis revealed that two basic ecDNAs might fuse together through recombination, and additional focal deletion may generate new subspecies with more complicated structures.^53^ Stronger evidence comes from a longitudinal study comparing ecDNA architectures in paired tumor samples before and after disease progression in Barrett’s esophagus (BE) and associated esophageal adenocarcinoma (EAC).^7^ BE is a pre-cancerous lesion with a small chance of progression to EAC. ecDNAs carrying oncogenes known to drive EAC, such as *KRAS* ecDNAs, were exclusively found in the BE that eventually progressed to EAC but not in non-progressors. By calculating the amplicon similarity score and complexity score, it has been shown that ecDNAs from the same origin increase in copy number and structural complexity as BE progresses to EAC.^7^ These studies support the idea that ecDNAs can undergo structural rearrangement even after they are formed.

Besides being subject to structural rearrangement themselves, ecDNAs can also serve as substrates for chromosomal rearrangements through reintegrating into the chromosomes (Fig. 4b).^21,22,24,55^ DNA damage on ecDNAs and chromosomes can drive ecDNA reintegration. This mechanism has been demonstrated in HeLa cells with ecDNAs carrying the dihydrofolate reductase gene (*DHFR*), in which CRISPR-Cas9-linearized ecDNAs are found to integrate into the CRISPR cutting sites on the chromosome.^22^ Current evidence suggests that the outcome of ecDNA reintegration may be beneficial to cancer. For example, through allele analysis, ecDNAs are found to reintegrate into certain chromosomal regions in neuroblastoma, leading to genomic remodeling. Importantly, such reintegration events are significantly enriched in cancer-relevant genes, associated with either the repression of tumor-suppressing genes or the activation of tumor-promoting genes.^55^ This study highlights the potential clinical impact of ecDNA-driven genomic instability. It is worth mentioning that such a chromosomal reintegration may only be relevant if it confers fitness to the cells and results in clonal selection as demonstrated in neuroblastoma, where *MYCN* ecDNA clustered reintegration is associated with worse patient survival.^55^

Lastly, recurrent mutations may further increase ecDNA sequence diversity. Mutational signature analysis revealed that more than 30% of ecDNAs have one or more associated kataegis events (clustered hypermutations) both in methotrexate-induced *DHFR* ecDNA in vitro and pan-cancer genomic research with clinical samples, dominated by APOBEC3 patterns.^22,56^ Chromothripsis can give rise to ecDNAs (which will be discussed in the later section), and chromothripsis-accompanied kataegis has been observed in shattered dicentric chromosomes after the telomere crisis.^57^ Notably, previous studies have shown that APOBEC-mediated clustered mutations can occur in close proximity to double-strand break sites.^58,59^ Pan-cancer genomic analysis has also revealed that 47% of APOBEC-mediated kataegis occurs near the breakpoints of the seismic amplifications, including ecDNA amplicons.^60^ These studies suggest that clustered mutations might be induced before or at the moment of self-ligation of DNA segments during ecDNA formation. However, another study reported that only ~7.2% of kataegis occurs early in the evolution of ecDNAs, whereas the majority of kataegis events probably occur after clonal amplification by recurrent APOBEC3 mutagenesis.^56^ In addition, kataegis events are enriched within ecDNA regions with known cancer-associated genes.^56^ Thus, kataegis may promote ecDNA diversity from ecDNA formation to ecDNA positive selection. While current studies have unraveled some mysteries regarding mutational events on ecDNAs, many questions remain unsolved: Is the mutation rate in ecDNAs higher than in chromosomes? Does kataegis in ecDNAs promote cancer evolution? Does kataegis in ecDNAs impact chemotherapy and targeted therapy responses? Can we leverage kataegis in ecDNAs to direct immunotherapy?

## Challenges and opportunities in treating ecDNA-driven cancer

### ecDNA imposes a clinical challenge

ecDNA is prevalent in human cancer. The first systematic pan-cancer investigation has estimated that ecDNA is present in nearly half of all cancer types and up to one-third of all cancer patients throughout their disease, while it was not found in matched blood and adjacent tissues.^4^ This study also revealed that ecDNA is associated with shorter survival across multiple cancers, even after adjusting for tissue type.^4^ Subsequent studies focused on specific cancer types, such as small cell lung cancer,^27^ neuroblastoma,^61^ medulloblastoma,^26^ and hepatocellular carcinoma,^54^ have similarly demonstrated that ecDNA is linked to poorer clinical outcomes.

**Fig. 5:**
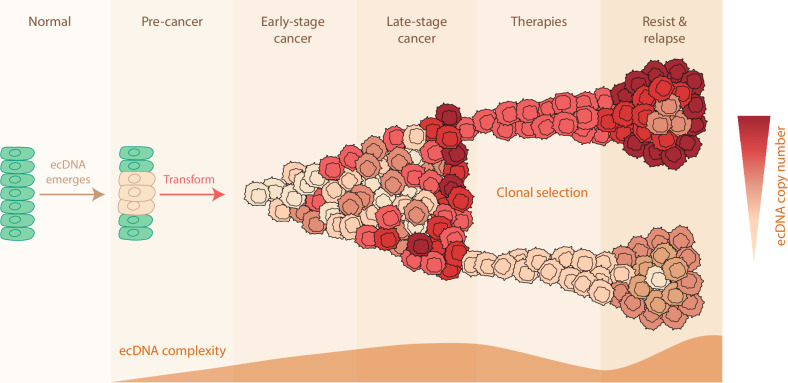
ecDNA in tumor formation, evolution, and drug resistance. ecDNAs may emerge before tumor formation, and subsequently drive malignant transformation. Along with tumor evolution, ecDNA copy number, complexity, and heterogeneity can increase. Under selective pressure of therapies, specific subpopulations of tumor cells with optimal ecDNA composition survive, rendering drug resistance and relapse.

A debate has since been raised: Is ecDNA a genuine cancer driver promoting early malignant transformation, or is it just a manifestation in late-stage cancer due to genomic instability? This appears to be a “chicken-and-egg” dilemma, but it turns out that both arguments are true (Fig. 5). As previously discussed, a longitudinal, case-control study in BE and associated EAC has shown that ecDNAs occur in the pre-cancerous lesions that eventually progressed to cancer.^7^ More importantly, ecDNAs were not found in pre-cancerous lesions that did not transform, even with long-term follow-up.^7^ Although further prospective studies are still required to clarify the causality between ecDNA and malignant transformation, it has provided strong evidence showing that ecDNAs can be generated preceding tumor formation. Another piece of evidence comes from a preprint study modeling ecDNA formation via the Cre-loxP system.^62^ The Cre recombinase can induce the circularization of a DNA segment flanked by two loxP sites with the same orientation. It has been shown that Cre-loxP-generated *MDM2* ecDNA enables malignant transformation in mouse embryonic fibroblasts driven by over-expression of the *HRAS* G12V mutant.^62^ While it has not been directly demonstrated that ecDNA alone is sufficient to drive transformation, applying this technology to generate specific ecDNAs in an appropriate tissue and developmental context is a promising way to address this question.

The most well-known challenge brought by ecDNA is therapy resistance. This was first documented nearly half a century ago when it was discovered that ecDNA amplification of the *DHFR* gene is responsible for methotrexate resistance.^63,64^ Methotrexate is an antimetabolite of folic acid that binds DHFR to inhibit the conversion of dihydrofolate into tetrahydrofolate, which is required for nucleotide synthesis. As a potent gene amplification mechanism, ecDNA can raise the *DHFR* copy number to an enormously high level, which, in turn, produces a massive amount of DHFR protein to dilute the effect of methotrexate. However, the rapid fluctuation in ecDNA copy number presents an even greater challenge. ecDNAs segregate randomly and create a heterogeneous pool of cancer cells containing diverse ecDNA species and subspecies of various copy numbers. This rich repertoire of heterogeneity provides adaptive fitness under the selective pressure of therapy.^65^

In certain contexts where high oncogenic output is beneficial, cancer cells with high ecDNA copy numbers are selected (Fig. 5). One of the most well-articulated examples is the acquired cross-resistance in small cell lung cancer driven by ecDNAs with *MYC* family paralogs.^37^ While most small cell lung cancer cases are initially chemosensitive, they often develop cross-resistance to multiple chemotherapy regimens upon relapse.^66,67^ A study comparing patient-derived xenografts derived before and after treatment has shown that chemotherapy can induce the formation of ecDNAs, including those carrying *MYC*.^37^ When chemoresistant xenografts are challenged by chemotherapies, DNA damage is selectively observed in the tumor cell population with low *MYC* ecDNAs. Consequently, tumor cells with high *MYC* ecDNAs are selected, leading to an increase in the overall *MYC* copy number over time.^37^ This study provides a direct, single-cell observation of the adaptive advantage conferred by ecDNA heterogeneity. A similar observation has been made in melanoma patients, in which mitogen-activated protein kinase (MAPK) inhibitors select for ecDNAs carrying resistance genes, such as *BRAF*.^24^

Conversely, tumor cell populations with low ecDNAs may be advantageous under different therapy pressures, especially with targeted therapy (Fig. 5). While clinical evidence is currently limited, experimental data using glioblastoma patient-derived cells have shown that the epidermal growth factor receptor (EGFR) kinase inhibitor erlotinib can deplete *EGFR* ecDNAs.^21^ Once the targeted drug loses its target, cancer cells become resistant. However, upon drug withdrawal, *EGFR* ecDNAs rapidly re-emerge within a few cell doublings.^21^ Given the fast kinetics of ecDNAs and their remarkable contributions to therapy resistance, it will be necessary to monitor ecDNA dynamics in clinical practice.

The understanding of ecDNA has been expanded with the discovery that ecDNA may carry immunoregulatory genes, such as PD-L1 in HPV fusion ecDNA and those identified in BE.^7,8^ This has sparked a growing interest in exploring how ecDNA-driven cancer may interact with the tumor microenvironment. A transcriptomic study based on bulk tumor RNA sequencing from The Cancer Genome Atlas (TCGA) suggests that cancers bearing ecDNA may exhibit an “immune-cold” phenotype. This is characterized by reduced immune cell infiltration and lower expression of antigen-presenting molecules compared to ecDNA-negative tumors.^68^ In line with this, another study using a machine-learning approach on transcriptomic data from TCGA has similarly discovered that ecDNA-positive cancers downregulate immunomodulatory processes, including lymphocyte activation, leukocyte chemotaxis, and cytokine production, which are integral to many facets of the cancer immune response.^69^

However, our knowledge of the detailed immunosuppressive landscape in ecDNA-positive cancers and the exact mechanism that drives the immune-cold phenotype is still limited. Studies so far are based on bulk-cell sequencing data, making it difficult to deconvolute cell composition and crosstalk within the tumor microenvironment. Furthermore, experimental interrogation is lacking; therefore, the causality between the ecDNA-positive tumors and the immune-cold phenotype is still unclear. More specifically, it is unclear whether ecDNA-bearing tumors can actively shape their tumor microenvironment or whether the immune-cold microenvironment favors the formation of ecDNAs in cancer cells. Applying single-cell and spatial genomic technologies to ecDNA mouse tumor models will open a new opportunity to understand the interplay between tumors with ecDNA and their microenvironment. This could potentially lead to significant advancements in our understanding of ecDNA biology, particularly its role in tumor development and progression, as well as provide new insights into devising immunotherapy approaches for ecDNA-driven cancers.

### Emerging thoughts on ecDNA-directed therapy

ecDNA has become a novel target for cancer therapy due to its unique role in cancer biology. First, ecDNA has shown pivotal roles in cancer development and therapy resistance. Second, ecDNA is exclusively present in cancerous and pre-cancerous but not in normal tissues. Third, targeting the protein products encoded by ecDNAs has proven challenging due to the dynamic nature and heterogeneity of ecDNA.^15,21^ More compellingly, targeting the oncogene-carrying ecDNA will make many currently undruggable targets actionable. While experimentally depleting ecDNA via CRISPR cutting is possible in cell culture,^15^ it is hard to imagine translating this approach into gene therapy for patients, considering the ongoing structural evolution of ecDNA in tumors. Current research endeavors focus on attacking ecDNA during both its formation and maintenance.

#### Targeting ecDNA formation

Mutational signature analysis on paired patient-derived xenografts, both before and after treatment, has unequivocally demonstrated that chemotherapy can induce the formation of ecDNAs and foster resistance.^37^ This finding prompts the question of whether the inhibition of ecDNA formation could serve as a feasible therapeutic option. Therefore, a comprehensive understanding of the pathogenesis mechanism of ecDNA is essential for effective prevention of ecDNA formation.

**Fig. 6:**
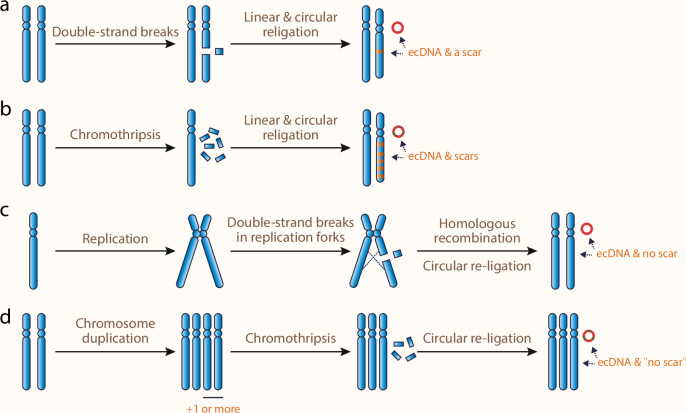
Scar-prone and scar-free ecDNA formation. **a** ecDNA formation with a scar on the chromosome. A chromosome undergoes mild DNA damage, such as two DNA double-strand breaks within a certain distance, releasing a linear DNA fragment. The broken chromosome and the free DNA fragment are re-ligated via end-joining individually, forming a circular ecDNA and a repaired chromosome with a focal deletion (scar). **b** ecDNA formation with multiple scars on the chromosome. A chromosome undergoes pulverization during chromothripsis, resulting in many DNA fragments. Some fragments ligate with each other to form ecDNA, while others reassemble into a heavily rearranged chromosome with multiple scars. **c** ecDNA formation without a scar on the chromosome. If mild DNA damage occurs in the replication forks during the S phase, the broken chromosome can be repaired by homologous recombination, leaving no scar on the chromosome while an ecDNA forms. **d** Another possible way to generate ecDNA without leaving a scar on the chromosome. Whole-chromosome or whole-genome duplication occurs before one of the chromosomes shatters. While the broken chromosome is depleted, ecDNA can arise from the fragmentized DNA, resulting in no visible scar on the chromosome.

In theory, ecDNA formation requires the initial breaks in its chromosome ancestors, resulting in the release of DNA segments that are subsequently re-ligated into a circular form. This process of cut-and-re-ligation explains why the presence of ecDNAs is often associated with deletion events in the original chromosomes (Fig. 6a). This phenomenon has been documented via southern blot, FISH, and sequencing in multiple cancer types.^70–72^ Experimental modeling of DNA double-strand breaks has confirmed that it is mechanistically possible. CRISPR-liberated chromosomal DNA segments can be self-ligated and form circular ecDNA, which can then be selected under appropriate selection pressure.^15,73^

While CRISPR models ecDNA formation from a mild focal deletion, ecDNA can also originate from more severe genomic instability contexts. For example, ecDNAs can arise from chromothripsis, a process where a chromosome mis-segregates and shatters into small pieces through multiple mechanisms, such as TREX1-mediated DNA resection,^74^ as well as pathological DNA damage responses via base excision repair^75^ and Fanconi anemia pathway.^76^ The shattered pieces are then re-ligated through DNA repair (such as non-homologous end-joining).^77,78^ During the re-ligation, centromere-less DNA fragments may be circularized to form ecDNA, resulting in complex rearrangements on both the derivative chromosome and ecDNA (Fig. 6b).^22,77,79^ In a pan-cancer study using the TCGA dataset, signatures of chromothripsis were detected in 36% of ecDNA amplicons and over 50% of samples with ecDNA,^4^ highlighting a significant role of chromothripsis in ecDNA formation.

However, certain studies have also shown that the presence of ecDNA does not necessarily correlate with a loss of heterozygosity.^72,80^ This observation suggests a copy-and-re-ligation hypothesis, in which DNA damage occurs in a replication fork, and the focally resected DNA scar is repaired by homologous recombination (Fig. 6c).^80^ However, although mechanistically possible, it still lacks evidence to support that the copy-and-re-ligation process naturally occurs and gives rise to ecDNA.

Alternatively, the cut-and-re-ligation model can still explain the scar-less ecDNA formation. If a whole-chromosome or whole-genome duplication event precedes the focal deletion, and the broken chromosome is then depleted due to processes like mis-segregation, the final outcome will appear as if there is no scar on the chromosome (Fig. 6d). This hypothesis is quite plausible because the formation of ecDNA is associated with the loss of function of *TP53*.^7,69^ A longitudinal study in BE and associated EAC shows that *TP53* deficiency precedes the formation of ecDNA.^7^ A TCGA pan-cancer analysis further shows that *TP53* is the only gene whose mutations are significantly higher in ecDNA-containing tumors.^69^ Therefore, when the compromised *TP53* primes genomic instability, such as whole-genome doubling,^81^ scar-less ecDNA formation is possible under the cut-and-re-ligation context.

After DNA segments are liberated from chromosomes, how are they re-ligated to form ecDNA? Breakpoint analyses suggest the existence of multiple possible pathways, including classic and alternative non-homologous end-joining and homologous recombination, with frequency varying among cancer types and treatment conditions.^72,80,82^ In line with these studies, inhibition of DNA-PKcs or PARP, both of which are important regulators of DNA repair pathways, can decrease the frequency of ecDNA formation under the context of chromothripsis.^22,82,83^ However, it remains an open question as to whether ecDNA formation can bypass such inhibition via alternative pathways. Although targeting multiple pathways is possible, we posit that any strategies aimed at targeting ecDNA formation must be carefully tailored to the specific cancer type and treatment context to mitigate the risk of toxicity from unnecessary medical interventions.

#### Targeting ecDNA maintenance

When a tumor is already ecDNA-positive at diagnosis, it is more rational to eliminate ecDNA rather than prevent ecDNA formation for therapy. If critical mechanisms of ecDNA maintenance are disrupted, it is possible to deplete ecDNA in cancer.

How is ecDNA maintained within the cancer genome? Similar to chromosomal DNA, ecDNA relies on at least three essential processes for their maintenance: (1) replication before mitosis to maintain their copy number; (2) segregation to ensure inheritance among daughter cells; (3) repair upon DNA damage. Additionally, genomic stability is overseen by the cell cycle checkpoint and innate immunity surveillance.^84–86^ Given that ecDNA is a manifestation of genomic instability, there is growing interest in understanding how ecDNA may evade the checkpoints of the cell cycle and innate immunity for their maintenance.

Certain small circular DNA species, such as plasmids and bacteriophage genomes, can duplicate through rolling-circle replication (RCR).^87^ However, chromatin-containing ecDNA of megabase sizes in cancer is unlikely to be replicated by RCR, because mammalian cells lack essential genes to initiate RCR, such as *RepB* and *RepC*. Early investigation using Okazaki fragment hybridization mapping indicated that ecDNA contains a bidirectional replication origin.^88^ In addition, differential staining of sister chromatids using thymidine analogs has shown that ecDNA replicates once and only once during the S phase in a semi-conservative manner, showing double-minute morphology.^29,89^ These observations suggest that ecDNA replication may employ machinery similar to that used for chromosomal DNA. Consequently, the opportunity to target ecDNA may not lie in the DNA synthesis process itself, but rather in how ecDNA and chromosomal DNA mitigate replication stress, which is a common feature in cancer.^90^

DNA replication, while it might seem like a simple “copy-paste” process, often encounters many obstacles, such as DNA secondary structures, DNA–RNA hybrids, and limited nucleotide availability.^91^ These obstacles lead to replication stress, a common feature of the cancer genome.^90^ It has been proposed that ecDNA is more susceptible to replication stress, because hydroxyurea, a replication stress inducer that inhibits ribonucleotide reductases to reduce the nucleotide pool, can induce ecDNA loss in cell culture.^92,93^ However, it remains unclear whether ecDNA particles are more sensitive to nucleotide shortage, or whether the presence of ecDNA renders cancer cells more vulnerable to hydroxyurea. A recent study argues that ecDNA suffers from a higher degree of replication-transcription conflict due to their elevated transcriptional activity.^94^ This, in turn, makes ecDNA-bearing cancer cells more vulnerable to the inhibition of CHEK1, a critical checkpoint protein in DNA damage response and cell cycle.^94–96^

Other aspects of the ecDNA maintenance mechanisms are largely unexplored. A TCGA pan-cancer transcriptomic study reveals specific upregulation of DNA double-break repair pathways in ecDNA-bearing tumors, including classic and alternative end-joining, single-strand annealing, and homology-directed repair pathways.^69^ While this finding has not been experimentally validated, it highlights the importance of understanding how ecDNAs are repaired in different damage contexts and whether there is an ecDNA-specific repair mechanism that could be exploited for pharmacological targeting. Previously, several studies showed that treatments with DNA-damaging agents, such as hydroxyurea and radiation, can lower ecDNA levels.^97,98^ These findings suggest the potential feasibility of targeting the mechanisms involved in ecDNA damage repair and maintenance. However, because these studies induced DNA damage globally that was not specific to ecDNA, it is challenging to deconvolute ecDNA-specific DNA damage response.

Mechanistic insights into ecDNA segregation also remain limited. While ecDNA has been observed to “hitchhike” on mitotic chromosomes to segregate into daughter nuclei,^15,48^ the factors mediating ecDNA–chromosome interaction during mitosis have not been identified. Theoretically, the contact (but not hybridization) between double-strand DNA molecules requires protein intermediaries, as the negative charges from the phosphate groups on the DNA backbone would otherwise repel such an interaction; unless DNA and histone modifications (such as methylation and acetylation, which are also protein-mediated processes) happen to create a favorable electrostatic interaction environment perfectly. Early studies on viral episomes, which are circular, extrachromosomal viral DNAs that attach to mitotic chromosomes for segregation, have identified many proteins coded by the virus genome involved in the attachment of episomes to mitotic chromosomes.^99–102^ These viral proteins bind to episomes and interact with host proteins on mitotic chromosomes, enabling episome segregation. However, most ecDNAs do not encode any foreign proteins other than those found in the cancer genome (except for the possibility of ecDNA–virus hybrids), it remains unclear what proteins are recruited to mediate the interaction between ecDNAs and mitotic chromosomes. Identifying these factors will provide a better understanding of ecDNA segregation and offer new therapeutic targets.

Research into ecDNA-specific targeting is still in its infant stage, and emerging new ecDNA targeting strategies beyond tackling ecDNA formation and maintenance, such as targeting ecDNA’s mobility, have been proposed.^103^ We anticipate that it will become one of the most important directions in the next decade. While advances in modeling and screening technologies have facilitated the study of ecDNA-specific maintenance mechanisms, we should be cautious when translating our knowledge yielded from basic science research into clinically actionable targets and trials. Cancer therapy is always a trade-off between efficacy and toxicity. This principle is equally applicable to ecDNA-directed therapy. The degree to which a target or a mechanism is specific to ecDNAs, as opposed to chromosomes, will be a critical factor in determining the therapeutic window.

### Clinical diagnosis for ecDNA: Where are we now?

With growing evidence supporting that ecDNA can serve as a predictive biomarker for clinical outcomes, such as malignant transformation, patient survival, and therapy response as discussed above, and ecDNA-directed clinical trials are on the horizon, there is a pressing need for the development of more cost-effective tools for ecDNA detection.

In our previous review of the current ecDNA detection toolbox, we highlighted that direct visualization of ecDNAs in cells at metaphase is considered the gold standard.^3^ The development of computer-assisted image analysis software, such as ECdetect^14^ and ecSeg,^104^ has facilitated automatic ecDNA detection. Even when metaphase chromosome preparation is not available, such as in formalin-fixed paraffin-embedded tissue samples,^105^ there are ongoing efforts to use machine learning technologies to detect ecDNAs by recognizing the signal pattern from FISH in interphase nuclei. Given that cytogenetics has been a standard clinical practice for decades, the imaging-based method is one of the most promising routes to be integrated into the existing clinical pipeline. However, as we have previously discussed, this approach suffers from low throughput, necessitating the development of more advanced, high-throughput technologies. Furthermore, the selection of a FISH probe requires prior sequence information, unless recurrent ecDNAs that contribute to a specific clinical outcome have been identified, such as those amplifying *MYC* and paralogs in chemoresistant small cell lung cancer.^37^

In comparison, sequencing-based methods offer a higher throughput. Currently, paired-end whole-genome short-read sequencing remains the most accessible method, as it does not require any special modifications of the experimental protocol for ecDNA detection. The ecDNA information is retrieved by computational analysis using the AmpliconArchitect software, which can analyze the structure of an amplicon by interval search, structural variation detection, and breakpoint graph construction and visualization.^106^ AmpliconArchitect is the only tool so far to extract ecDNA information from short-read WGS. As such, WGS is more feasible for automation, from sample processing to data interpretation. However, AmpliconArchitect cannot distinguish between ecDNA and ecDNA-originated homogeneously staining regions derived from ecDNA reintegration to the chromosome. The reintegration often maintains the fine structure of multiple copies of ecDNA. Therefore, it is challenging to find a sufficient number of reads to support reintegration junctions in short-read sequencing.^107^ And while CIRCLE-seq is designed to enrich circular DNA for sequencing by digesting linear DNA,^55,108^ its time-consuming procedure limits its clinical application.

Recent research efforts have been increasingly focused on delineating the heterogeneity of ecDNA by leveraging single-molecule detection technologies. One such approach involves the use of nanopore long-read sequencing to reconstruct complex ecDNA architectures.^109,110^ When a sequencing read is sufficiently long to cover many unique breakpoints of an ecDNA, it becomes possible to retrieve information about structural variations at the single-ecDNA resolution. An alternative strategy, known as CRISPR-CATCH, employs the Cas9–sgRNA complex to linearize ecDNA and physically separate it with a pulse-field electrophoresis gel for downstream sequencing.^6^ These methods have collectively revealed the enormous heterogeneity of ecDNA, which may reshape our understanding of how diverse ecDNA contributes to clonal expansion, as well as how transcriptional circuits are differentially rewired in ecDNA particles.

The rapid advancement of modern technologies for ecDNA detection has facilitated ecDNA basic science research. However, the translation into a clinically actionable approach is yet to be realized. An optimal clinical diagnostic tool should at least meet the following criteria:

1. Highly sensitive and specific, particularly for clinical specimens with limited cell input, such as liquid biopsies.
2. Can be seamlessly incorporated into the current clinical pipeline, as employing a new technology is a major commitment.
3. Cost-effective and affordable to patients with cancer.

At present, no technology can be directly implemented and promoted in clinical practice. For instance, even though the cost of next-generation sequencing has significantly reduced, it remains a significant financial burden to patients and the healthcare system.^111^ This does not even account for the subsequent ecDNA analysis, which is still computationally expensive. Regarding imaging methods, current cytogenetic techniques require sufficient cell number input. Especially in the preparation of metaphase spreads, a substantial number of cells are discarded from analysis due to unpredictable morphology and poor separation of chromosomes and ecDNAs. Hence, the ongoing technological development in the field must address these issues and design diagnostic tools that are more sensitive, specific, affordable, and rapid, especially those that can be integrated into the existing clinical workflow.

## Outlook

**Fig. 7:**
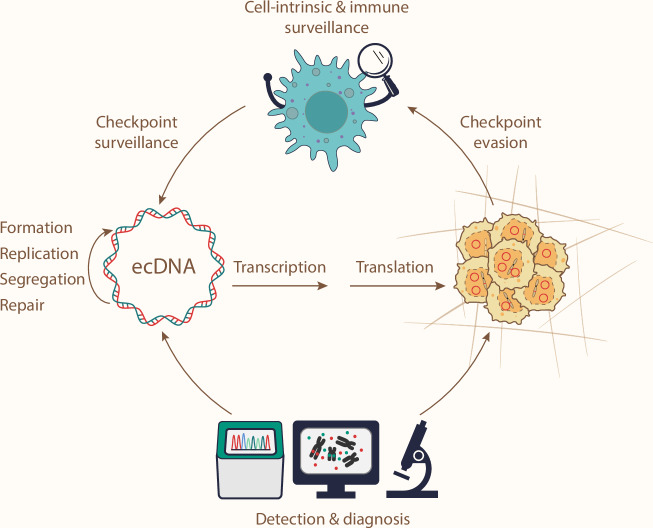
ecDNA-centered cancer biology and therapeutic vulnerabilities. ecDNA drives cancer transformation and progression, highlighting ecDNA as a therapeutic target. Opportunities to target ecDNA lie in its formation and maintenance mechanisms. In addition, understanding how cell-intrinsic processes and immune surveillance sense ecDNAs, and how ecDNA tumors evade these checkpoints, could further unlock therapeutic potential. Finally, ecDNA detection and diagnostic tools are required for ecDNA-directed clinical practices.

ecDNA presents a grand challenge to the clinical treatment and management of cancer. It is remarkable to witness that ecDNA resurfaced in the limelight in the last decade, despite being observed over half a century ago. Previous efforts have highlighted the importance of ecDNA in cancer biology and pioneered a suite of tools for its study. We foresee a future where ecDNA-centric cancer clinical trials, diagnostics, therapies, and management strategies are within reach in the coming decade (Fig. 7).

While ecDNA research is advancing rapidly, uncharted territory remains in ecDNA biology. It is worth considering: if we replaced every instance of “DNA” with “ecDNA” in a biology textbook, would all the statements still hold true? Particularly, the maintenance mechanisms for ecDNA in the cancer genome are largely unknown, yet this knowledge is essential for developing effective ecDNA-targeting strategies. For example: How do ecDNA replicate? Do ecDNA respond to replication stress and DNA damage differently? What proteins mediate the interaction between ecDNA and chromosome during mitosis? Can cell-intrinsic and immune checkpoints sense ecDNA?

Furthermore, ecDNA brings a new challenge to technological advancement, particularly in the realm of genomics. The high copy number and heterogeneity of ecDNA render data from many bulk-cell sequencing and profiling approaches difficult to interpret. The prospect of assessing ecDNA at single-cell and even single-molecule resolution offers a promising avenue to address these issues. This would enable a comprehensive re-examination of ecDNA biology to unravel key differences between ecDNA and chromosomal DNA, in terms of their functions and maintenance mechanisms. By harnessing this knowledge, we aim to identify an optimal therapeutic window to treat ecDNA-driven cancer, potentially in an oncogene-agnostic fashion.
